# Association between Body Fatness and Vitamin D_3_ Status in a Postmenopausal Population

**DOI:** 10.3390/nu12030667

**Published:** 2020-02-29

**Authors:** Héctor Vázquez-Lorente, Jorge Molina-López, Lourdes Herrera-Quintana, Yenifer Gamarra-Morales, Beatriz López-González, Elena Planells

**Affiliations:** 1 Department of Physiology, School of Pharmacy, Institute of Nutrition and Food Technology “José Mataix”, University of Granada, 18071 Granada, Spain; hectorvazquez@ugr.es (H.V.-L.); lourdesherrera@correo.ugr.es (L.H.-Q.); beatrizlogo@yahoo.es (B.L.-G.); elenamp@ugr.es (E.P.); 2 Department of Physical Education and Sports, Faculty of Education, Psychology and Sports Sciences, University of Huelva, 21007 Huelva, Spain

**Keywords:** vitamin D, vitamin D_3_, vitamin D_2_, menopause, UHPLC, BMI

## Abstract

Vitamin D is a micronutrient that plays a key role in phosphocalcic metabolism. The postmenopausal population presents a risk of deficiency in this vitamin due to hormonal alterations which, in the case of obesity, would be exacerbated. The objective was to assess the status of vitamin D in a postmenopausal population and determine the relationship of 25-hydroxivitamin D [25(OH)D] and its metabolites with anthropometric parameters. The study included 78 healthy postmenopausal women aged from 44 to 76. The nutrient intake assessment was carried out using the 24 h reminder (R24h). 25(OH)D was analyzed using ultra-high-performance liquid chromatography (UHPLC). A total of 80% and 68% of the women studied did not reach sufficient values of 25(OH)D and 25-hydroxivitamin D_3_ [25(OH)D_3_], respectively, which was inversely correlated with Body Mass Index (BMI) (*r* = −0.25, *p* = 0.04), hip perimeter (*r* = −0.26 and r = −0.24, all *p* < 0.05), arm circumference (*r* = −0.29, *p* = 0.01) and fat mass (*r* = −0.28 and *r* = −0.26, all *p* < 0.05). 25(OH)D_3_ is the metabolite that contributed most to this association. In conclusion, 25(OH)D_3_ levels are related to anthropometric parameters in the postmenopausal women in this study, confirming insufficient status in the majority of the population. Approach strategies are necessary to correct and avoid this risk in order to ensure future quality of life.

## 1. Introduction

Vitamin D is a fat-soluble vitamin that enters the body through exposing the skin to sunlight and through food and dietary supplement intake. Vitamin D is formed by the sum of the metabolites vitamin D_2_ and vitamin D_3_, with vitamin D_3_ being most important and plentiful in humans because it comes from more food sources than vitamin D_2_; in addition, it is the only metabolite that is synthesized in the epidermis. For vitamin D to become biologically active, it must first be hydroxylated in the liver to 25-hydroxyvitamin D (25-OH-D), which is the metabolite used to assess a subject’s vitamin D status, and then in the kidneys to 1,25-dihydroxyvitamin D (1,25-(OH)_2_-D), which is its active form [1]. Vitamin D deficiency is a worldwide public health problem related to skeletal and non-skeletal problems [2] due to nutritional deficits, liver and/or kidney failure, resistance to the action of vitamin D [3], and a low exposure to sunlight and the use of sunscreen [4]. Likewise, the theory that genetics has an impact on vitamin D deficiency is gaining strength [5]. On the other hand, vitamin D is well known for its role in regulating phosphocalcic metabolism [6]. A vitamin D deficiency causes a decrease in the intestinal absorption of calcium (Ca), reducing its status and triggering the release of Parathyroid Hormone (PTH), levels of which are inversely proportional to the levels of 25(OH)D [7]. Therefore, the optimal serum concentration of 25(OH)D is defined as the concentration that suppresses the maximum release of PTH, being a measure of vitamin D deficiency and vitamin D toxicity [8].

Vitamin D deficiency is a widespread problem in the overweight population [9]. Obese subjects usually have lower sun exposure, reduced skin biosynthesis, or some intrinsic factor related to obesity, such as the volumetric dilution of vitamin D in adipose tissue [10]. Therefore, obesity can also be a factor to consider when establishing vitamin D recommendations [1]. Coupled with an increased low-grade inflammatory state [11] (which seems to be more pronounced in women), this makes imposes higher vitamin D requirements in obesity [12]. Although the increase in body weight may have a protective effect on bone fractures, the scientific rationale showed some potential explanations that link obesity with increased fracture risk with reduced 25-hydroxy-vitamin D concentrations [13]. In addition, the extent to which serum concentrations are sensitive to change as a result of weight loss has not been determined [9]. The decrease in the concentration of 25-OH-D may be due to its contribution to muscle metabolism, recommending special attention to supplementation in overweight postmenopausal women who do physical activity [14].

The problem of a lack of vitamin D is also very common in menopause, which is the transitioning phase of a woman’s life from the reproductive to the non-reproductive period. In this stage, there will be endocrine changes due to decreased ovarian activity, biological changes due to decreased fertility, and clinical changes resulting from changes in the menstrual cycle [15], with a wide variety of symptoms [16]. During menopause, women will have thinner skin and a lower capacity to produce vitamin D, in addition to a decrease of intestinal absorption of vitamin D and a decrease in vitamin D hydroxylation in the liver and kidneys. These metabolic problems will be accompanied by a tendency towards limited outdoor activity and a lower dietary intake of vitamin D [17]. On the other hand, an association has been found between vitamin D and sex hormones in postmenopausal women, where the reduction of estrogen levels, as well as other hormonal changes, causes a tendency to develop low levels of vitamin D [18]. These hormonal alterations can cause musculoskeletal, metabolic and cardiovascular conditions and can affect mental health, all of which being related to vitamin D deficiency [19,20]. In studies carried out of menopause, low levels of vitamin D were associated with a higher frequency of clinical fractures and low bone mass [21], in addition to the percentage of postmenopausal women with osteoporosis being higher in those with vitamin D deficiency [22]. During menopause, it has been shown that women may be particularly susceptible to the consequences of vitamin D deficiency, since a decrease in bone mineral density (BMD) and lean mass, as well as an increase in fat mass, occurs [23] in this period of life as a result of the decrease in estrogen levels.

The scientific literature reflects that the levels of 25(OH)D are inversely associated with BMI in postmenopausal women, though the role of different vitamin D metabolites in BMI and other anthropometric parameters is not yet clarified [1,24]. Therefore, our study aimed to assess the status of vitamin D in a postmenopausal population and determine the relationship between 25(OH)D and its metabolites with anthropometric parameters in a postmenopausal population from Granada.

## 2. Materials and Methods 

### 2.1. Subjects and Study Design

The present study is a cross-sectional design in which the study population was 78 postmenopausal women from Granada, aged between 44 and 76 years. The inclusion criteria were based on acceptance to participate in the study after being informed about it and presenting amenorrhea for at least 12 months. The exclusion criteria were being a perimenopausal woman, undergoing hormone replacement therapy, refusal to participate in the study for various reasons, the presence of pathologies that may affect the absorption of nutrients, as well as being in a situation of disease that could alter the biochemical parameters analyzed. The confidentiality of all the data used and collected has been guaranteed at all times, complying with the principles of the Declaration of Helsinki and the approval by the Ethics Committee of the University of Granada (149/CEIH/2016).

### 2.2. Data Collection

All recorded data were obtained through the use of questionnaires that reflected information on personal data, sociodemographic aspects, nutrient intake and anthropometric measurements, as well as other aspects, such as an adequate diagnosis of the postmenopausal situation, smoking habits and physical activity.

### 2.3. Intake Rating

Dietary nutrient intake was quantitatively assessed using an R24h, taking into account a holiday and two non-holidays. Recall accuracy was recorded with a set of photographs of prepared foods and dishes that are frequently consumed in Spain. The food intake assessment was converted to both energy and nutrients, determining the adequacy of the macronutrient and micronutrient intake according to the recommended daily amount (RDA) of that nutrient for the Spanish population of women within the age range included in our study using the Nutriber^®^ software 1.1.5. version, Barcelona, Spain. 

### 2.4. Anthropometric Assessment

Height and total body weight were measured according to the international standards for anthropometric assessment. Height was assessed with a stadiometer (Seca, model 213, range 85 to 200 cm; precision: 1 mm; Hamburg, Germany). Body composition measurements were taken for all participants, obtaining muscle mass, fat mass, weight, percentage of body fat and BMI with bioelectrical impedance (Tanita MC-980 Body Composition Analyzer MA Multifrequency Segmental, Barcelona, Spain). In addition, the arm, waist and hip perimeters were measured using a height rod with a 0.01 cm range of error. BMI was calculated as weight (kg) divided by the square of height (m^2^). The study group was classified according to their BMI in the following groups: Normal weight < 27 kg/m^2^; overweight-obesity > 27 kg/m^2^ [25,26], corresponding to the median of the data. Waist circumference was measured at the midpoint between the lower margin of the least palpable rib and the top of the iliac crest [27]. Hip circumference was measured at the widest portion of the buttocks, with the tape parallel to the floor [28]. Middle arm circumference was measured in the right arm at the midpoint between the tip of the olecranon and the acromion, with the arm hanging loosely [29]. A global adiposity factor for further analysis was calculated through the mean z-score values for BMI, arm circumference and hip perimeter.

### 2.5. Measurement of Biochemical Parameters

A blood extraction was performed and was centrifuged at 4 °C for 15 min at 3000 rpm, extracting the plasma. Ca was determined through flame atomic absorption spectrophotometry (FAAS, Perkin Elmer^®^ Analyst 300 model, Berlin, Germany), and P was determined using the Fiske-Subbarow colorimetric method (Thermo Scientific, Rockford, IL, United States). PTH and osteocalcin were measured using EIA by colorimetric method (ECLIA, Elecsys 2010 and Modular Analytics E170, Roche Diagnostics, Mannheim, Germany). The remaining biochemical parameters, such as glucose, urea, uric acid, creatinine, triglycerides, total cholesterol, HDL and LDL cholesterol, total proteins, transferrin, albumin, homocysteine, bilirubin, transaminases, LDH and PCR levels were determined in the analysis unit at the Virgen de las Nieves Hospital, Granada, Spain.

#### Analytical Determination of Vitamin D by UHPLC

For protein precipitation, 200 µL of plasma was taken in an Eppendorf, to which 20 µL of internal standard (IS) (Sigma Aldrich, St. Louis, MO, USA) (0.5 µg/mL) and 500 µL of acetonitrile were added. This was taken to the plate shaker at an amplitude of 3, form 2 for 1 min and dried with N_2_. It was then centrifuged at 10000 × *g* for 15 min at 4 °C. The supernatant was collected in another Eppendorf, discarding the rest. For extraction phase, the supernatant was mixed with 100 µL of water and 200 µL of ethyl acetate, then mixed in vortex 30 s. Subsequently, it was centrifuged at 3000× *g* for 5 min. The supernatant was collected and the previous step was repeated with the precipitate, collecting the supernatant again and combining it with the previous one. Finally, the supernatants were dried with N_2_. For derivatization step, a solution of 4-Phenyl-3H-1,2,4-triazole-3,5(4H)-dione (PTAD) (Sigma Aldrich, St. Louis, MO, USA) in acetonitrile (0.5 mg/mL) was prepared and mixed in vortex. Then, 50 µL of PTAD was added to both samples and standards and was taken to the plate shaker for 1 h at room temperature covered with aluminum foil. Finally, the samples were transferred to vials for UHPLC and 50 µL of water was added. They were covered with aluminum foil and stored in the freezer at −20 °C to puncture in UHPLC. For preparing the calibration line, increasing concentrations of 1 ppb, 2 ppb, 5 ppb, 10 ppb, 25 ppb, 50 ppb and 100 ppb of the standards (25-OH-D_3_), (25-OH-D_2_), (1,25-(OH)_2_-D_3_), (1,25-(OH)_2_-D_2_), vitamin D_2_ and vitamin D_3_ (Sigma Aldrich, St. Louis, MO, USA) were added to the vials, as well as 20 µL of the IS. The mixture was dried with N_2_ and finally derivatized and stored at −20 °C for further analysis.

Samples were analyzed using the Waters Acquity UHPLC I-Class System chromatograph (Waters, London, UK). The column used was an Acquity UHPLC BEH C18 ™ column 2.1 × 50 mm, 1.7 µm at room temperature. The injection volume of the sample was 10 µL. The mobile phase of channel A was water with 50 mM of ammonium formate and that of channel B was methanol. The analysis time was 8 min and the flow rate 0.4 mL/min. The detector was a Waters XEVO-TQ-XS Triple Quadrupole Low Resolution Spectrometer. The source of ionization was electrospray ionization with positive ionization. The desolvation temperature was 600 °C and the desolvation gas flow rate was 500 L/h. The source temperature was 150 °C and the gas flow rate of the cone 150 L/h. The biochemical values of 25(OH)D obtained were classified according to the reference values of 25(OH)D and 25(OH)D_3_ in plasma. These are sufficiency >30 ng/mL, insufficiency 20–30 ng/mL, and deficiency <20 ng/mL for 25(OH)D [30] and sufficiency >20 ng/mL and deficiency <20 ng/mL for 25(OH)D_3_ [31,32].

### 2.6. Statistical Analysis

The statistical analysis was performed using SPSS software (IBM SPSS statistics, Version 22.0, Armonk, NY, USA: IMB Corp.). Descriptive statistics were used for data expression, indicating the results of numerical variables as the arithmetic mean, standard deviation (X ± SD), and standard error of the mean (SEM), and the results of the categorical variables were expressed in frequencies (%). As a previous step to the execution (or not) of a parametric model, the hypothesis of the normal distribution was accepted using the Kolmogorov–Smirnov test. For the comparative analysis based on the BMI categories (BMI < 27 kg/m^2^ and BMI > 27 kg/m^2^), the *t*-test for parametric samples was used. Likewise, Student’s *t*-test and the analysis of variance test (ANOVA) were used to compare the status of both vitamins according to the BMI. Linear regression analysis was used to estimate the degree of association between the vitamin D and D_3_ status, and the anthropometrical parameters.

## 3. Results

Table 1 shows the intake values of macronutrients and the main micronutrients involved in phosphocalcic metabolism categorized by BMI < 27 kg/m^2^ and BMI > 27 kg/m^2^. With reference to anthropometrical parameters, it should be noted that the percentage of body fat, especially in the group with the highest BMI, was much higher than its reference value (32%). The other anthropometric parameters are approximately within the reference values, although they were more suitable in the group with the lowest BMI. Regarding energy intake, the diet was hypocaloric in both groups, but the group with the highest BMI had a higher hypocaloric tendency. In general, the population followed a slightly hyperproteic diet, and while the rest of the variables were within the reference values, except for vitamin D, levels were below the reference values in both groups (<40% RDA). The average carbohydrate (CHO) intake was lower in the group with the highest BMI (*p* < 0.05).

Table 2 reflects the biochemical parameters classified by BMI categories <27 kg/m^2^ and >27 kg/m^2^. The data obtained were framed within the reference values, reflecting that the postmenopausal population of the study was healthy.

Table 3 shows the average values for the most relevant parameters of phosphocalcic metabolism in the postmenopausal population classified by BMI categories. From a general view, 80% of the general population did not reach sufficient vitamin D status, and about half had deficiency levels of <20 ng/mL. In the case of vitamin D_3_, 80% of the population was deficient and 32% had sufficient status. In addition, the decrease in 25(OH)D and 25(OH)D_3_ as the BMI increased was statistically significant (*p* < 0.05), an association that 25(OH)D_2_ did not experience.

**Figure 1:**
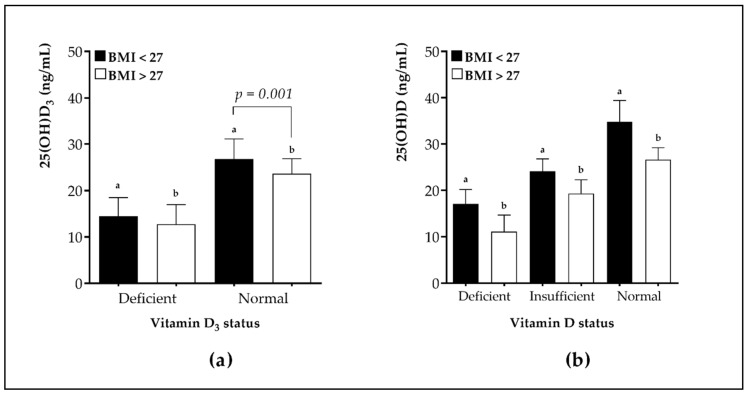
(**a**) Mean levels of 25(OH)D_3_ metabolite in BMI > 27 and BMI < 27 (expressed in kg/m^2^) groups, each divided into subgroups based on vitamin D_3_ status (Subgroup I: sufficiency > 20 ng/mL; subgroup II: deficiency < 20 ng/mL). (**b**) Mean levels of 25(OH)D metabolite in BMI > 27 and BMI < 27 (expressed in kg/m^2^) groups, each divided into subgroups based on the vitamin D status (Subgroup I: sufficiency > 30 ng/mL; subgroup II: insufficiency 20–30 ng/mL; subgroup III: deficiency < 20 ng/mL). ^a^ statistically significant differences (*p* < 0.05) in vitamin D or D_3_ status when BMI < 27 kg/m^2^. ^b^ statistically significant differences (*p* < 0.05) in vitamin D or D_3_ status when BMI < 27 kg/m^2^.

Additionally, Figure 1 shows the average levels of 25(OH)D and 25(OH)D_3_ metabolite classified by BMI and vitamin D and D_3_ status. The intragroup analysis revealed that postmenopausal women with a normal vitamin D_3_ status had significantly lower levels for this metabolite when the BMI was greater than 27.

Table 4 shows Pearson’s bivariate correlations of 25(OH)D and 25(OH)D_3_ levels with the anthropometric parameters analyzed in our study. A statistically significant inverse association was found between vitamin D and anthropometric parameters in general, such as BMI, arm and hip perimeter, as well as fat mass. This correlation was maintained when performing the bivariate analysis with the 25(OH)D_3_ metabolite. In addition, there was a statistically significant inverse correlation between 25(OH)D_3_ and categorized BMI, with this difference being more marked in the group with the highest BMI, hip perimeter and fat mass.

**Figure 2:**
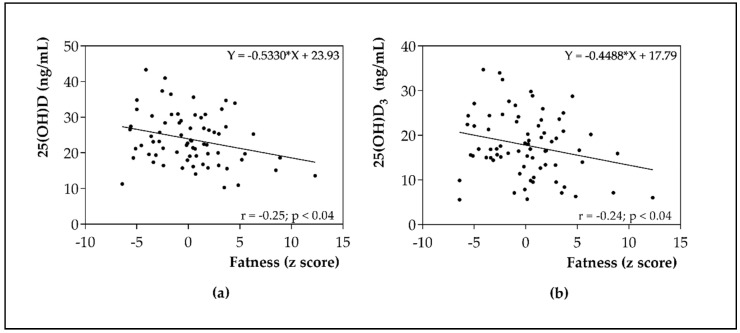
(**a**) Pearson’s bivariate correlation of 25(OH)D with fatness as z score; (**b**) Pearson’s bivariate correlation of 25(OH)D_3_ metabolite with fatness as z score.

Finally, Figure 2 shows the bivariate correlation analysis between anthropometric parameters expressed as fatness z-score and 25(OH)D and 25(OH)D_3_ metabolite levels. The results showed an inverse relationship between the predominance of fat and the levels of 25(OH)D and 25(OH)D_3_ (*p* < 0.05).

## 4. Discussion

One of the most important changes during the menopausal stage refers to the changes in body composition parameters mainly due to hormonal alterations during this stage. Previous evidence in the postmenopausal population showed an inverse association between vitamin D status and BMI. The role of anthropometric parameters as well as BMI on the status of vitamin D_3_ in this population is not clarified enough, and results demonstrate that a BMI cut-point of 30 kg/m^2^ does not appear to be an appropriate indicator of true obesity status in post-menopausal women [26]. In this line, Banack et al. [26] indicated that BMI cut points should potentially be replaced by 26.5 kg/m^2^ or 27.1 kg/m^2^; therefore, this study established it as 27 kg/m^2^, also based on the recommendations for an aged postmenopausal population [25]. The main findings of the present study showed that 80% of the general population did not have a sufficient vitamin D status (<30 ng/mL). Similarly, 68% of the population was deficient for 25(OH)D_3_ metabolite. The relationship between body composition through BMI and 25(OH)D levels revealed that those women with higher BMI (BMI > 27 kg/m^2^) presented lower status of that vitamin. The same results were observed for 25(OH)D_3_ levels (*p* < 0.05), with higher levels being observed in those with a lower BMI and fat mass. However, when the comparative analysis was carried out according to BMI for 25(OH)D_2_ metabolite, this statistically significant difference was not observed. On the other hand, no statistically significant correlation was observed between either 25(OH)D or its metabolites and age or the other parameters of phosphocalcic metabolism when categorized in the groups obtained according to BMI.

In our study, vitamin D intake was slightly low, not taking into account the role of the sun on vitamin D input. This trend coincides with another Spanish postmenopausal population, in which 96% of the women studied had Ca and vitamin D intakes lower than the RDA, highlighting the need to take measures aimed at protecting the bone health of the Spanish female population [33]. This pattern of intake deficit has already been repeated in other parts of the world, such as in the study by Macdonald et al. [34] conducted in a population of 3113 women in the United Kingdom, in which vitamin D intake was 4.2 µg/day; therefore, vitamin D intakes are far below what is required in latitudes that are different to those of Spain as well. Vitamin D intake should be emphasized for all latitudes, especially for those women living in latitudes with limited sun exposure [35] where it has become very common to supplement with vitamin D in the postmenopausal population [36]. In a study of vitamin D supplementation in the postmenopausal population, Zhao et al. [37] observed that subjects who began a vitamin D supplementation trial with a low serum vitamin D status during a cold season were more sensitive to vitamin D supplementation compared to subjects who started during a hot season and had elevated baseline levels of serum vitamin D.

In relation to the levels of 25(OH)D and its metabolites, a high prevalence of vitamin D deficiency was found in our population, which corresponds to a greater or lesser extent with other populations, such as that in the study performed by Arévalo et al. [38], in which the Argentine postmenopausal population showed a 27% vitamin D deficiency and 29% vitamin D insufficiency, finding a negative association with age. Stewart et al. [39] assessed a large-scale sample of postmenopausal women that included 18 countries worldwide. They found that there was a high prevalence of vitamin D deficiency in all countries studied, placing a special emphasis on the fact that 64% of the postmenopausal women with osteoporosis had insufficient levels of 25(OH)D. In the study by Li et al. [40] performed on Chinese postmenopausal women, approximately 72% of the women had a vitamin D deficiency. In this study, serum vitamin D levels were not correlated with BMI or fat mass, contradicting the results of our study. On the other hand, other authors, such as Shirazi et al. [41], found a positive association between 25(OH)D_3_ serum levels and age, phosphorus (P), Ca, and a high intake of vitamin D. One possible reason for the observed results that contradict our own is that in the present study, the older women in this cohort consume relatively more vitamin D.

In terms of the rest of the phosphocalcic metabolism parameters, authors such as Zhang et al. [42] state that serum P levels decrease progressively with age in postmenopausal women. In our study, P levels were within normal values, possibly due to a diet adequate in P. On the other hand, we observed that osteocalcin levels were higher as age increased (*r* = 0.28, *p* = 0.01). In the study by Alissa et al. [17], vitamin D intake and BMI were associated with low levels of osteocalcin; however, the same association with vitamin D and BMI was not found in our study. Other studies found an association between phosphocalcic metabolism parameters, such as P and PTH, and anthropometric parameters, like the study performed by Billington et al. [43], in which the authors maintain that serum phosphate from the postmenopausal population was inversely correlated with weight, BMI and fat mass. Bolland et al. [44] also found that PTH was positively correlated with body weight, regional and total fat mass and body fat percentage, but was negatively correlated with vitamin D. Similarly, we observed the same positive association between fat mass and PTH (*r* = 0.32, *p* = 0.005), although we did not find the direct relationship of this hormone with vitamin D status. Another study by Macdonald et al. [34] reflects that obese subjects had a lower vitamin D status and higher concentrations of PTH compared to non-obese subjects. Finally, Khadka et al. [45] found that vitamin D and Ca were negatively correlated with the year of menopause onset, suggesting medical supervision of hormonal changes and periodic dosing of vitamin D and Ca in postmenopausal women to reduce the bone health problem.

In our study, an inverse association was found between vitamin D status and fat mass. However, the weakness of this association might be explained by the presence of numerous factors which modulate fat mass, such as ethnicity, age [46], sun exposure habit, diet, and the season of the year in which the samples were taken, which can mean that even though a woman has a high fat mass, its influence on vitamin D status can lose strength [47,48]. In our study, due to the limited sample size, we could not control for some of these confounding factors. Authors such as Lucas et al. [49] maintain that 25(OH)D levels are inversely related to fat mass and positively related to physical activity. However, we have not been able to demonstrate the positive relationship of vitamin D status with physical activity, probably due to low physical activity patterns in our postmenopausal women. Authors like Vuksanovic et al. [50] stated that visceral fat is more harmful than subcutaneous fat, emphasizing that women with high amounts of visceral fat have low serum 25(OH)D levels. Therefore, it would be necessary to assess the location of body fat and its influence on vitamin D status for future studies. Similarly, we observed how the hip perimeter was negatively correlated with both 25(OH)D and 25(OH)D_3_ levels, confirming the relationship of those vitamins with parameters that are usually used to determine the central obesity. Abboud et al. [51] found lower vitamin D status in those subjects with a high hip perimeter; nevertheless, they did not reflect the role of 25(OH)D_3_ metabolite in this association. According to our results, a decrease in body fat is accompanied by an increase in the status of vitamin D_3_. However, Holecki et al. [52] performed an intervention to reduce body weight in menopause, achieving a significant decrease in body fat and concluding that in obese subjects the serum concentration of 25(OH)D_3_ was significantly lower before and after intervention. In addition, in our study we found an inverse relationship between 25(OH)D and 25(OH)D_3_ status with BMI. Sousa-Santos et al. [53] found this correlation between BMI and vitamin D status, although they also failed to discern between the different metabolites of vitamin D. Other authors, such as Liu et al. [54] and Kocot et al. [55], support the role that BMI has on vitamin D_3_ status (*r* = 0.09, *p* = 0.01), although they did not assess whether this association was influenced by total body fat as we demonstrated in our results. The prevalence of obesity, associated with a reduced quality of life, morbidity and mortality, underscores the need for a food reeducation program during the postmenopausal period [56]. Therefore, to improve the status of vitamin D, regular use of low doses of supplemental vitamin D_3_ [57,58] in case of deficiency, compliance with vitamin D and Ca RDAs, and maintaining an adequate weight is recommended in postmenopausal population [59].

## 5. Conclusions

Our data reflect that the high prevalence of vitamin D and vitamin D_3_ deficit observed in our postmenopausal population is generally correlated with BMI, in addition to anthropometric parameters such as hip and arm circumference and fat mass defined as fatness. According to our data, it seems that 25(OH)D_3_ is the vitamin D metabolite that is most closely related to the anthropometric parameters studied. Therefore, nutritional assessment and vitamin D_3_ supplementation policies are proposed, as well as related healthy habits to improve the status of vitamin D in at-risk groups, such as postmenopausal women, to optimize their quality of life.

## Figures and Tables

**Table 1:** Anthropometric parameters and nutrient intake values.

(Characteristics)	BMI < 27 (kg/m^2^) (*n* = 39)	BMI > 27 (kg/m^2^) (*n* = 39)	*p* Value	(Reference Values)
	(Mean ± SD)	(Mean ± SD)		
Age (years)	57.7 ± 8.2	58.5 ± 8.5	0.7	-
BMI (kg/m^2^)	23.3 ± 2.6	30.5 ± 3.1	<0.001	22–27
Blood pressure ^1^	1.5 ± 1.4	1.9 ± 1.4	0.2	-
Physical exercise ^2^	1.2 ± 0.9	1.2 ± 0.8	0.9	-
Arm perimeter (cm)	27.9 ± 1.9	31.7 ± 2.7	<0.001	<30
Waist perimeter (cm)	80.0 ± 8.9	97.5 ± 9.5	<0.001	<90
Hip perimeter (cm)	98.3 ± 6.2	113 ± 8.7	<0.001	<110
Waist/hip ratio	0.8 ± 0.1	0.8 ± 0.1	0.005	<0.80
Body fat (%)	33 ± 5.6	41 ± 3.7	<0.001	23–31
Fat mass (kg)	20.1 ± 5.1	32.1 ± 6.7	<0.001	-
Muscle mass (kg)	37.9 ± 7.7	45.8 ± 4.7	<0.001	-
Energy intake (kcal)	1500 ± 300	1300 ± 400	0.06	2000
CHO intake (g/day	160 ± 37.5	140 ± 45.0	0.04	275
Protein intake (g/day)	63.6 ± 14.1	59.5 ± 16.3	0.2	50
Fat intake (g/day)	61.6± 17.1	56.6 ± 23.3	0.3	70
Cholesterol intake (mg/day)	170 ± 73.8	163 ± 63.5	0.7	<300
Fiber intake (g/day)	16.8 ± 6.7	15.1 ± 9.3	0.3	>25
Ca intake (mg/day)	841 ± 262	800 ± 255	0.7	800–1000
P intake (mg/day)	1045 ± 321	1031 ± 292	0.8	800
Vitamin D intake (µg/day)	3.7 ± 3.7	3.3 ± 2.7	0.6	<10

^1^ Blood pressure shows the values 0 = optimal, 1 = normal, 2 = normal–high, 3 = grade 1 hypertension; ^2^ Physical exercise covers the values 0 = non-sedentary exercise, 1 = <1 h/day, 2 = 1–2 h/day, 3 = <2 h/day. Values are expressed as mean ± standard deviation. The fourth column shows the statistical significance after applying the comparison of means for not related samples.

**Table 2:** Biochemical parameters.

(Characteristics)	BMI < 27 (kg/m^2^) (*n* = 39)	BMI > 27 (kg/m^2^) (*n* = 39)	*p* Value	(Reference Values)
	(Mean ± SD)	(Mean ± SD)		
Glucose (mg/dL)	88.2 ± 17.5	100 ± 13.5	0.04	70–110
Transferrin (mg/dL)	300 ± 53.3	300 ± 37.4	0.7	200–360
Prealbumin (mg/dL)	24.1 ± 5.8	26.2 ± 4.1	0.1	20–40
Albumin (mg/dL)	4.5 ± 0.3	4.4 ± 0.2	0.4	3.5–5.2
Homocysteine (µmol/L)	11.9 ± 4.7	11.4 ± 4.9	0.6	<13
Creatinine (mg/dl)	0.7 ± 0.1	0.7 ± 0.1	0.4	0.5–0.9
^1^ LDH (U/L)	200 ± 26.4	192 ± 58.6	0.2	110–295
Urea (mg/dL)	34.0 ± 9.3	35.0 ± 8.9	0.6	10–50
Uric acid (mg/dL)	4.2 ± 1.2	4.6 ± 0.9	0.2	2.4–5.7
Triglycerides (mg/dL)	106 ± 63.4	100 ± 72.6	0.8	50–200
HDL cholesterol (mg/dL)	70.2 ± 18.3	63.3 ± 11.5	0.06	40–60
LDL cholesterol (mg/dL)	129 ± 32.7	127 ± 30.3	0.7	70–190
Total cholesterol (mg/dL)	223 ± 34.8	218 ± 34.2	0.5	110–200
^2^ GOT (U/L)	22.0 ± 5.0	22.5 ± 7.7	0.7	<37
^3^ GPT (U/L)	18.0 ± 7.5	21.2 ± 12.6	0.2	<41
^4^ GGT (U/L)	18.0 ± 9.9	21.8 ± 18.2	0.2	11–50
^5^ CRP (mg/L)	1.9 ± 9.9	0.3 ± 0.2	0.3	0.02–5
Total bilirubin (mg/dL)	0.5 ± 0.2	0.5 ± 0.12	0.2	0.10–1.2
Total proteins (g/dL)	7.1 ± 0.5	7.1 ± 0.5	0.7	6.6–8.7

Values are expressed as the mean ± standard deviation. ^1^ LDH: Lactate Dehydrogenase, ^2^ GOT: Glutamic oxaloacetic transaminase, ^3^ GPT: Glutamic pyruvic transaminase, ^4^ GGT: Gamma-glutamyltransferase, ^5^ CRP: C-reactive protein.

**Table 3:** Phosphocalcic metabolism parameters.

Parameter	BMI < 27 (kg/m^2^) (*n* = 39)	BMI > 27 (kg/m^2^) (*n* = 39)	*p* Value	(Reference Values)
	(Mean ± SD)	(Mean ± SD)		
25(OH)D (ng/mL)	26.1 ± 7.3	21.9 ± 6.6	0.01	30–100
25(OH)D_3_ (ng/mL)	19.5 ± 7.4	16.1 ± 6.5	0.04	>20
25(OH)D_2_ (ng/mL)	5.8 ± 3.9	5.7 ± 2.3	0.9	>10
Ca (mg/dL)	9.3 ± 0.5	9.1 ± 0.4	0.2	8.6–10.2
P (mg/dL)	3.5 ± 0.5	3.4 ± 0.5	0.4	2.7–4.5
Osteocalcin (ng/mL)	14.2 ± 10.6	16.2 ± 9.1	0.4	15–46
PTH (pg/mL)	53.2 ± 17.5	58.9 ± 28.2	0.3	20–70

PTH: Parathyroid hormone.

**Table 4:** Matrix for correlation coefficients (r) showing the simple linear relationship between anthropometrical characteristics, vitamin D and vitamin D_3_.

	Categorized BMI (kg/m^2^)	25(OH)D (ng/mL)	25(OH)D_3_ (ng/mL)
Uncategorized BMI (kg/m^2^)	*r* = 0.8	*r* = −0.25	*r* = −0.2
	*p* < 0.001 *	*p* = 0.04 *	*p* = 0.09
Categorized BMI (kg/m^2^)	-	*r* = −0.29	*r* = −0.24
		*p* = 0.01 *	*p* = 0.04 *
Arm circumference (cm)	*r* = 0.7	*r* = −0.24	*r* = −0.2
	*p* < 0.001 *	*p* = 0.04 *	*p* = 0.1
Waist circumference (cm)	*r* = 0.6	*r* = −0.14	*r* = −0.11
	*p* < 0.001 *	*p* = 0.2	*p* = 0.3
Hip perimeter (cm)	*r* = 0.7	*r* = −0.26	*r* = −0.24
	*p* < 0.001 *	*p* = 0.03 *	*p* = 0.04 *
Waist/hip ratio	*r* = 0.3	*r* = 0.06	*r* = 0.06
	*p* = 0.005 *	*p* = 0.6	*p* = 0.5
Body fat (%)	*r* = 0.6	*r* = −0.2	*r* = −0.2
	*p* < 0.001 *	*p* = 0.1	*p* = 0.1
Fat mass (kg)	*r* = 0.7	*r* = −0.28	*r* = −0.26
	*p* < 0.001 *	*p* = 0.02 *	*p* = 0.03 *
Muscle mass (kg)	*r* = 0.5	*r* = −0.2	*r* = −0.15
	*p* < 0.001 *	*p* = 0.1	*p* = 0.2
25(OH)D (ng/mL)	*r* = −0.29	-	*r* = 0.90
	*p* = 0.01 *		*p* < 0.001 *
25(OH)D_3_ (ng/mL)	*r* = −0.24	*r* = 0.90	-
	*p* = 0.04 *	*p* < 0.001 *	

Matrix correlations are presented as correlation coefficients (*r*). * statistical significance = *p* < 0.05.
